# The Role of Sildenafil in Treating Brain Injuries in Adults and Neonates

**DOI:** 10.3389/fncel.2022.879649

**Published:** 2022-05-10

**Authors:** Ying Xiong, Pia Wintermark

**Affiliations:** ^1^Department of Anesthesiology, Zhongnan Hospital of Wuhan University, Wuhan University, Wuhan, China; ^2^Research Institute of the McGill University Health Centre, Montreal, QC, Canada; ^3^Division of Newborn Medicine, Department of Pediatrics, Montreal Children’s Hospital, Montreal, QC, Canada

**Keywords:** brain, neonate, neonatal encephalopathy, neuroprevention, neurorestoration, sildenafil

## Abstract

Sildenafil is a recognized treatment for patients suffering from erectile dysfunction and pulmonary hypertension. However, new evidence suggests that it may have a neuroprotective and a neurorestorative role in the central nervous system of both adults and neonates. Phosphodiesterase type 5—the target of sildenafil—is distributed in many cells throughout the body, including neurons and glial cells. This study is a comprehensive review of the demonstrated effects of sildenafil on the brain with respect to its function, extent of injury, neurons, neuroinflammation, myelination, and cerebral vessels.

## Introduction

Sildenafil is a highly potent selective inhibitor of phosphodiesterase type-5 (PDE5i); it regulates the second messenger cyclic guanosine monophosphate (cGMP) by inhibiting the effects of PDE5 that breaks down the phosphodiesteric bond of cGMP and hydrolyze cGMP into GMP. Since its launch in 1998, sildenafil has been used widely to treat erectile dysfunction (ED) for its recognized vasodilatatory and smooth muscle relaxant effects (Giuliano et al., 2010). Sildenafil also has been used as a treatment for pulmonary hypertension (PH) in adults (Galiè et al., 2015; Vitulo et al., 2017). Following its approval for the treatment of pulmonary hypertension in adults, the off-label use of sildenafil for treating pulmonary arterial hypertension in infants has increased (Hsieh et al., 2013) and has spread to neonates (Steinhorn et al., 2009; Pierce et al., 2021), which has shown that it also could be used safely with that population, with undeniable beneficial effects (Abman, 2007; Mukherjee et al., 2009; Steinhorn et al., 2009; El-Ghandour et al., 2020; He et al., 2021; Kamran et al., 2021).

Sildenafil has been shown to reach other body organs with demonstrated beneficial effects, such as improved cardiovascular function in fetal growth restricted offsprings (Terstappen et al., 2019), alleviated endothelial dysfunction in patients with diabetes (Desouza et al., 2002), and improved acute kidney injury in adults (Whitaker et al., 2013). With respect to the brain, sildenafil has been demonstrated to cross the blood-brain barrier (BBB) (Gómez-Vallejo et al., 2016) and to reach the central nervous system. PDE5—the target of sildenafil—has been described as being present in neurons and glial cells (astrocytes and microglias), as well as cerebrovascular endothelial cells (both endothelial and smooth muscle cells) (Teich et al., 2016). Also, the NO/cGMP signal pathway has been shown to regulate neurogenesis, neuroinflammation, myelination, synaptic plasticity, and cerebral blood flow in adults (Feil and Kleppisch, 2008; Raposo et al., 2013; Garthwaite et al., 2015). Thus, PDE5 has gained increasing attention as a potential therapeutic target for several adult central nervous system diseases, including adult stroke (Chen et al., 2017), multiple sclerosis (Pifarre et al., 2011), Alzheimer’s disease (Zuccarello et al., 2020), epilepsy (Tawfik et al., 2018), memory dysfunction (Sikandaner et al., 2017), and hepatic encephalopathy (Agusti et al., 2017). Through inhibiting this enzyme, sildenafil may exert neuroprotection and neurorestoration (Ribaudo et al., 2016; Nabavi et al., 2019).

Interestingly, to date, most studies that have shown the beneficial effects of sildenafil in the brain have been run with animal models of adult diseases, such as stroke, Alzheimer disease, and multiple sclerosis. However, only a few studies have tested its potential in the human adult brain. And only a few studies have highlighted the potential effects of sildenafil in the neonatal brain. Considering that this drug already has been used safely off-label with neonates to treat persistent pulmonary hypertension, it also may prove advantageous for treating their brain disorders. Recently, Zinni et al. (2021) reviewed the neuroprotective role of sildenafil. Our review discusses in detail its neurorestorative effects, including its impact on neurogenesis and myelination, as well as its neuroprotective role and its effects on brain function, infarct size, neuroinflammation, and angiogenesis (Table 1). Moreover, our review also discusses sildenafil’s mechanisms of action and its pharmacokinetics in adults and neonates. In addition, our review highlights the first evidence of similar beneficial effects on the injured brain of neonates.

**TABLE 1:** The role of sildenafil in adult and neonatal brain injury.

	Adult	Neonate
Function	**Motor function:** promote motor function recovery (Agusti et al., 2017) **Cognitive function:** improve spatial learning (Palmeri et al., 2013); memory retention (Cuadrado-Tejedor et al., 2011); object recognition (Venkat et al., 2019) **Emotional function:** improve anxiety-like behavior (Ozbeyli et al., 2015) **Other:** analgesia (Huang L. J. et al., 2009); anti-convulsant (Nieoczym et al., 2010)	**Motor function:** promote motor function recovery (Charriaut-Marlangue et al., 2014; Yazdani et al., 2016; Engels et al., 2017)
Infarct size	Increase cerebral blood flow level in ischemic penumbra area (Bednar, 2008); reduce infarct size, only 24 h post-injury (Chen et al., 2014)	Reduce infarct size (Moretti et al., 2016; Yazdani et al., 2016)
Neuron	**Neuroprotection:** Inhibit neuron death (Thakur et al., 2013; Díaz-Lucena et al., 2018; Thabet et al., 2021); reduce neurodegeneration (Ferreira et al., 2013); stabilize neuronal function (Cuadrado-Tejedor et al., 2011); promote synaptic plasticity (Uthayathas et al., 2013; Chen et al., 2014; Araújo et al., 2020); preserve axon ultrastructure (Nunes et al., 2012); **Neurorestoration:** Promote neurogenesis (Zhang et al., 2006, 2012; Santos et al., 2014); promote synaptogenesis (Zhang L. et al., 2005; Venkat et al., 2019)	Increase number of neurons (Yazdani et al., 2016) **Neuroprotection:** Prevent neuron death (Ozdegirmenci et al., 2010; Yazdani et al., 2021) **Neurorestoration:** Promote neurogenesis (Gómez-Pinedo et al., 2010; Engels et al., 2017; Yazdani et al., 2021)
Neuroinflammation	**Microglia:** Regulate the activation and polarization of microglia (Pifarré et al., 2014; Zhao et al., 2016; Agusti et al., 2017) **Astrocytes:** Regulate activation of astrocytes and formation of glial scar (Prado et al., 2013; Agusti et al., 2017; Hansson et al., 2018) **Leukocytes:** Regulate leukocytes infiltration (Chen et al., 2017); prevent the release of cytokines (Agusti et al., 2017)	Attenuate reactive gliosis (Järlestedt et al., 2010) **Microglia:** Regulate the activation and polarization of microglia (Moretti et al., 2016)
Myelination	**Myelination:** Prevent demyelination (Nunes et al., 2012, 2016b); promote remyelination (Pifarre et al., 2011) **Oligodendrocytes:** Inhibit apoptosis of OLs (Pifarré et al., 2014); promote pre-OLs maturation (Díaz-Lucena et al., 2018); enhance oligodendrogenesis (Zhang et al., 2012; Venkat et al., 2019)	**Oligodendrocytes:** enhance oligodendrogenesis (Shrivastava et al., 2012; Duy et al., 2015)
Angiogenesis	**Vascular:** Improve angiogenesis (Ding et al., 2008, 2011; Tawfik et al., 2018; Ibrahim et al., 2021); regulate hemodynamics (Zhang et al., 2006; Han et al., 2012; Kruuse et al., 2012); promote microvascular reactivity (Diomedi et al., 2005; Rosengarten et al., 2006; Lindberg et al., 2017) **Cerebral metabolism:** Increase cerebral oxygen metabolism (Sheng et al., 2017)	**Vascular:** Regulate hemodynamics (increase cerebral blood flow) (Moretti et al., 2016; Charriaut-Marlangue et al., 2014) **Cerebral metabolism:** Promote cerebral oxygenation (Nagdyman et al., 2006)

## Mechanisms of Action of Sildenafil

Mounting evidence has indicated that the NO-cGMP-PKG pathway is a central mechanism, interconnecting neuroinflammation, neurodegeneration, and cognitive disorders, which has led to an increased pharmaceutical interest in PDE5 as a promising therapeutic targets for neurological diseases in which these processes are involved. Upstream of cGMP, the amino acid L-arginine is converted by three possible varieties of the enzyme nitric oxide synthase (NOS) into nitric oxide (NO). NO is a small cell-permeable gas molecule that diffuses across the plasma membrane and activates soluble guanylyl cyclase (sGC). The latter may regulate synthesis of cGMP through converting guanosine 5’-triphosphate (GTP) into cGMP. The effects of cGMP are determined by three types of intracellular receptors: cGMP dependent kinases (PKG), ion channels regulated by cGMP, and PDEs regulated by cGMP.

**FIGURE 1:**
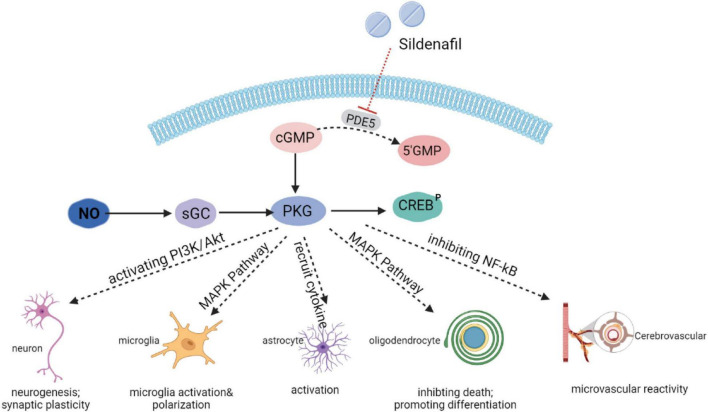
Mechanisms of action of sildenafil.

As a highly potent selective PDE5i, sildenafil has recently emerged as a therapeutic strategy of interest through an accumulation of cGMP and activation of the protein kinase G (PKG). Subsequently, PKG targets different pathways (Figure 1). Sildenafil has been found to enhance neurogenesis and synaptic plasticity through the activation of the PI3K/Akt pathway in adults (Peixoto et al., 2015) and neonates (Yazdani et al., 2021). In a mice model of autoimmune encephalomyelitis, sildenafil inhibited microglia activation and the death of oligodendrocytes through the mitogen-activated protein kinase (MAPK) signaling pathway (Duarte-Silva et al., 2018, 2021). PKG also increased the production of inflammatory cytokines [interleukin-1β (IL-1β), interleukin-6 (IL-6), and tumor necrosis factor alpha (TNF-α), etc.], which could have an impact on the activation of microglia and astrocytes (Nunes et al., 2016a). In addition, the NO-cGMP pathway inhibited the vascular nuclear factor-kappa B (NFκB) inflammatory activity, and increased microvascular reactivity (Spiecker et al., 1997).

## Sildenafil Use in Neonates

The European Medicines Agency (EMA) approved sildenafil for children at low doses (<2 mg/kg/day) and, in 2014, the Federal Drug Administration (FDA) indicated that the benefit-risk profile of sildenafil (up to 6 mg/kg/day) was acceptable in children 1 year of age and older if closely monitored (Abman et al., 2013). These recommendations were based on chronic use of sildenafil (6 mg/kg/day for ≥ 2 years) in children with pulmonary hypertension that was idiopathic or associated with congenital heart disease (Barst et al., 2012, 2014). More recently, the use of sildenafil has been tested in fetuses with severe intrauterine growth retardation (25 mg 3×/day between 22 and 29 weeks of gestation) with variable results (Sharp et al., 2018). However, none of these studies focused on the short-term administration of sildenafil to neonates with NE. Sildenafil treatment still remains off-label in neonates. Thus, a need exists to determine whether using sildenafil in these neonates has benefits (Wintermark, 2011; Kelly et al., 2017; Chakkarapani et al., 2021; He et al., 2021). Some recent reviews and case series with >140 neonates indicated a good safety profile for sildenafil (Samiee-Zafarghandy et al., 2014; Perez and Laughon, 2015), which was similar to available randomized trials (Baquero et al., 2006; Pierce et al., 2021). The only described side effect for neonates was a decrease in blood pressure, which typically occurred only with the first dose, and was easily controllable with volume expansion and/or inotropic support (Baquero et al., 2006; Steinhorn et al., 2009; Pierce et al., 2021), so trials of sildenafil in neonates with NE should be planned carefully, since they may be hemodynamically very unstable during the first days of life. Concurrently, sildenafil may improve the persistent pulmonary hypertension that often is present concomitantly in neonates with NE (Lapointe and Barrington, 2011; Giesinger et al., 2017) and thus prevent further injury secondary to impaired oxygenation.

## Effects of Sildenafil on Brain Function

### Adult Evidence

Sildenafil has been shown to have a beneficial effect on cognitive function in several animal models of adult diseases. For example, in a physiological mouse model of aging, sildenafil exerted neuroprotection against age-related cognitive dysfunction (Palmeri et al., 2013). Also, in a mouse model of Alzheimer’s disease, sildenafil administrated for 5 weeks improved spatial learning and memory retention (Cuadrado-Tejedor et al., 2011). Similarity, in a transgenic mouse model of amyloid deposition (Alzheimer’s disease), sildenafil acted as a memory enhancer *via* modulating memory storage, improving object memory and counteracting spatial learning impairment, both immediately during treatment and for a prolonged period beyond the drug administration (Puzzo et al., 2009). In a transgenic mouse model of Huntington’s disease, sildenafil treatment improved deficits in object recognition memory and passive avoidance learning (Saavedra et al., 2013). Furthermore, in stroke rat models, sildenafil administered for 7 consecutive days—starting either 2 h, 24 h, or 7 days after the stroke—promoted functional recovery measured by a foot fault test and an adhesive removal test 7 or 28 days after hypoxia-ischemia (Zhang et al., 2002, 2003). Sildenafil also restored motor coordination in beam walking, which implied a promotion of cerebellar function (Agusti et al., 2017) in rats with portacaval shunt (PCS). In both stressed and non-stressed animals, sildenafil markedly reduced serum cortisol levels (Socała et al., 2016), which could suggest protection against stress. Similarly, sildenafil was shown to improve cognitive function (measured by an object recognition test) and reduce anxiety-like behavior in sedentary rats exposed to acute stress exposure (Ozbeyli et al., 2015).

In addition, in a mouse model of neuropathic pain, sildenafil has been proposed as an analgesic, secondary to its antinociceptive effect by the induction of both GABAA and GABAB receptors and by increasing the threshold to pain (Huang L. J. et al., 2009). Sildenafil also has been shown to induce peripheral analgesia in a dose-dependent way (Jain et al., 2001). Finally, the anticonvulsant properties of sildenafil are still being debated, since sildenafil may have both pro- and anticonvulsant activity, depending on the experimental model of epilepsy, the animal species and the sildenafil dosage (Nieoczym et al., 2010; Tawfik et al., 2018). With respect to human patients, sildenafil has enhanced their ability to focus attention on streams of auditory stimuli (Schultheiss et al., 2001).

Thus, treatment with sildenafil may have an impact on cognitive function (spatial learning, memory retention, and object recognition), motor function (motor coordination), emotional function (anxiety-like behavior), analgesia, and seizures.

### Neonatal Evidence

Sildenafil’s potential effects on neonates have been tested using animal models after hypoxia-ischemia. Sildenafil decreased neurological deficits 20 days post-injury as measured by a gait analysis (Yazdani et al., 2016) and promoted motor functional recovery 7 days post-injury as measured by an open-field test (Charriaut-Marlangue et al., 2014) and 30 days post-injury as measured by an elevated plus maze (EPM) and wire hang test (Engels et al., 2017).

Clinical trials (NCT02812433 and NCT04169191) are ongoing to determine whether similar beneficial functional effects can be observed in human neonates after birth asphyxia.

## Effects of Sildenafil on Extent of Brain Injury

### Adult Evidence

In rat models of adult stroke, the effects of sildenafil on the extent of ischemic brain injury are still being debated. One study has found a clear reduction of the cerebral infarct volume 24 h post-injury, when a single intravenous or intraperitoneal dose of sildenafil (8–32 mg/kg) was given 2 h post-hypoxia-ischemia (Chen et al., 2014). However, in a rat model of embolic stroke, sildenafil treatment (10 mg/kg) started 24 h after injury and continued daily for 6 days after embolus placement did not significantly reduce the lesion size 6 weeks after injury (Li et al., 2007), even though it improved function and enhanced angiogenesis. In a rat model of middle cerebral artery (MCA) stroke, the administration of sildenafil for 1 week in escalating doses from 25 to 150 mg did not affect brain infarct size, even though it preserved synapse function and increased angiogenesis (Bednar, 2008). The individual variations in the severity of injury associated with the different animal models of adult stroke (Patel et al., 2014) and the different regimens (doses and duration) of the sildenafil treatments may explain the differences in outcomes.

### Neonatal Evidence

Several studies on the neonatal brain have now demonstrated that sildenafil treatment may reduce the extent of brain injury after hypoxia-ischemia. For example, Yazdani et al. (2016) found that *per os* treatment with sildenafil—started 24 h after hypoxia-ischemia at P10 and continued for 7 consecutive days (14 doses) —reduced the extent of injury 20 days post-injury and that higher doses (10 and 50 mg/kg) appeared to be more efficient than smaller doses (2 mg/kg). Charriaut-Marlangue et al. (2014) found that at postnatal day 7 (P7), a single dose of sildenafil that had been given intraperitoneally immediately after hypoxia-ischemia reduced brain injury, and the reduction was again dose-dependent—the 10 mg/kg was effective, but not the 5 mg/kg dose. Their follow-up study showed that this effect was not evident 72 h after hypoxia-ischemia, but was evident 8 days post-injury (Moretti et al., 2016). However, Engels et al. (2017) at P9, did not observe any improvement of brain injury with a single intraperitoneal dose of sildenafil 10 mg/kg given 2 h post-hypoxia-ischemia at P9, despite an observed functional improvement. Similar to adults, the effects of sildenafil may be dose- and duration-dependent.

## Effects of Sildenafil on Neurons

### Evidence in Adults

Sildenafil treatment appears to be neuroprotective. It reduces neuronal death by inhibiting apoptosis (Díaz-Lucena et al., 2018) and balances the degree of autophagy (Venkat et al., 2019). In a rat model of chronic cerebral hypoperfusion, sildenafil given for 10 consecutive days post-injury reduced neurodegeneration in both the CA1-CA4 striatum of the hippocampus and cerebral cortex, and preserved granular cells in the dentate gyrus 3 months post-injury (Ferreira et al., 2013). In a rat model of adult stroke, sildenafil administered for 1 day alleviated neuronal damage and synaptic structure degeneration (Chen et al., 2014).

Simultaneously, sildenafil appears to be neurorestorative and encourages recovery after injury by promoting neurogenesis, synaptogenesis, and synaptic plasticity, and by preserving axon ultrastructure. Sildenafil stimulates neurogenesis by promoting the proliferation of neural progenitor cells and neural stem cells and by inducing the maturation of immature neurons in the neural circuit (Zhang et al., 2012; Santos et al., 2014). Sildenafil also has been shown to enhance synaptogenesis (Zhang X. et al., 2005), especially around the ischemic boundary regions, and thus regulates synaptic plasticity (Uthayathas et al., 2013). This effect has been found not only in animal models of adult stroke, but also in animal models of adult neurodegenerative diseases. In a transgenic mouse model of Alzheimer’s disease, a 5-day treatment with sildenafil in 14-month-old mice increased synaptic function through the regulation of Arc proteins, which resulted in the restoration of spatial learning and memory retention (Cuadrado-Tejedor et al., 2011). A 14-day administration of sildenafil in a rat model of Huntington’s disease attenuated cell damage (Thakur et al., 2013). In a seizure model, sildenafil restored the normal hippocampal neuronal architecture and preserved the hippocampal neuronal cell count (Tawfik et al., 2018). In a vascular dementia model in aged rats, a 28-day treatment of sildenafil improved synaptic plasticity through upregulating synaptophysin (Venkat et al., 2019). Finally, in a cuprizone-induced rat model of multiple sclerosis, sildenafil treatment preserved axon ultrastructure (Nunes et al., 2012; Araújo et al., 2020).

Interestingly, the effects of sildenafil on neurons appear somewhat age-dependent. In Ding et al.’s (2008) study, the therapeutic response to sildenafil treatment was weaker and delayed after hypoxia-ischemia in aged rats compared to younger adult rats, which reflected poorer brain plasticity in aged rats This difference in brain plasticity could be explained, at least partly at the molecular level, by the fact that the function of the NO/cGMP signaling pathway—that regulates angiogenesis, neurogenesis, axonal outgrowth, and synaptic plasticity during development and in adulthood—is age-dependent, with observed impairment in this pathway with advanced age (Chalimoniuk and Strosznajder, 1998).

In a safety study of human adult patients with stroke, sildenafil was found to be safe (Silver et al., 2009).

### Evidence in Neonates

After birth, the neonatal brain still undergoes a significant degree of neuronal, synaptic, and angiogenic growth (Jensen, 2006). Axonal growth, neuronal dendritic arborization, and the establishment of synaptic contacts, myelination, and glial differentiation are among the many processes still occurring during the first postnatal weeks of rodents (Andersen, 2003) and during the first years of life in humans (Giedd et al., 1999; Andersen, 2003). Considering that the effects of sildenafil on neurons appear somewhat age-dependent, its beneficial effects may be even more marked in the injured maturing neonatal brain. Interestingly, Gómez-Pinedo et al. (2010) have demonstrated that rats whose cGMP was blocked during gestation presented with a reduced differentiation of stem cells in neurons and an increased differentiation in non-neuronal cells, and this effect normalized when sildenafil was given at the same time.

Far fewer research studies have investigated the effects of sildenafil on the neurons in the neonatal brain after hypoxia and ischemia. In an animal model of fetal ischemia, sildenafil treatment of mothers proved to be anti-oxidative, which could prevent neuronal death (Ozdegirmenci et al., 2010). Yazdani et al. (2016) demonstrated that *per os* treatment with sildenafil—started 24 h after hypoxia-ischemia at P10 and continued twice a day for 7 consecutive days (14 doses)—could cause a significant increase in the number of neurons near the infarct boundary 20 days post-injury (Vannuci rat model at P10), with the higher doses being the most efficient (10 and 50 mg/kg). Their follow-up study demonstrated that sildenafil may be neuroprotective in the neonatal brain by preventing apoptosis, but also neurorestorative by promoting neurogenesis (Yazdani et al., 2021). Engels et al. (2017) found an increased number of immature neurons in the ipsilateral subventricular zone and striatum after a single intraperitoneal dose of sildenafil (10 mg/kg) given 2 h post-hypoxia-ischemia at P9.

## Effects of Sildenafil on Neuroinflammation

### Evidence in Adults

In an effort to cope with pathogens, toxins, insult, trauma, and degeneration, the central nervous system can mount an elaborate response called *neuroinflammation*, which is characterized by the leakage of the blood-brain barrier (BBB) (Becher et al., 2017) and the orchestrated actions of microglia, astrocytes, cytokines release, recruited blood leukocytes, and infiltrated macrophages from the periphery (Xiong et al., 2016). Russo and McGavern (2016) have suggested that some degree of inflammation may be necessary in the acute stage of brain injury to clear damage and set the stage for remodeling efforts. However, excessive or persistent neuroinflammation may be harmful and may further contribute to the progression of brain injury in both adults (Agusti et al., 2017) and neonates (Järlestedt et al., 2010). Drug modulating neuroinflammation may exert different/opposite effects on neuroinflammation, depending on the maturity of the brain and the timing of when the drug is administrated after injury.

Sildenafil has been demonstrated to have potent anti-inflammatory properties throughout the body: i.e., in animals with severe acute pancreatitis (Fang et al., 2020), bronchial asthma (Laxmi et al., 2019), intestinal ischemia, and reperfusion injury (Moore et al., 2019); and in humans with type 2 diabetes (Schlindwein et al., 2010), diabetic cardiomyopathy (Nurnberg et al., 2002), and heart failure. In the brain, sildenafil appears to play a role in modifying the inflammatory balance. In microglia cell culture, sildenafil inhibits microglia activation. In astrocyte cells culture, sildenafil downregulated several inflammatory receptors, including the Toll-like receptor 4 (TLR4), the substance P receptor (NK-1), and the protease-activated receptor 2 (PAR-2) (Hansson et al., 2018). In a rat model of hepatic encephalopathy, sildenafil mitigated inflammation *via* inhibiting the activation of microglia and astrocytes, and by preventing the release of cytokines (Agusti et al., 2017). In a model of traumatic brain injury, sildenafil inhibited the activation of microglia (Prado et al., 2013). In a rat model of irradiation-induced brain injury, sildenafil showed anti-inflammatory and antioxidant properties through the modulation of the NO/tetrahydrobiopterin (BH4) pathway (Thabet et al., 2021). In addition, in a model of experimental autoimmune encephalomyelitis (EAE), sildenafil appeared neuroprotective by promoting the switch of microglia from the proinflammatory M1 phenotype to the anti-inflammatory M2 phenotype (Pifarré et al., 2014), which reduced the infiltration of CD4+ T lymphocytes and the production of IL-17 and TNFα (Araújo et al., 2020), and activated autophagy (Duarte-Silva et al., 2021).

### Evidence in Neonates

In the neonatal brain, the immaturity of the immune system, the ongoing developmental neuronal apoptosis, and the different balance between pro- and anti-oxidant enzymes appear to create a window of increased susceptibility to neuroinflammation secondary to hypoxic-ischemia injury (Vexler and Yenari, 2009). Shrivastava et al. (2012) observed a glial/inflammatory response with an activation of microglia/macrophages and astrocytes and a recruitment of neutrophils from 3 h after hypoxia-ischemia at P7 up to 100 days after HI in the ipsilateral hemisphere, and from 3 to 72 h in contralateral hemisphere. Järlestedt et al. (2010) found that the attenuation of reactive gliosis, which appeared protective in the adult model of focal brain ischemia, did not reduce the volume of infarct after neonatal hypoxia-ischemia, although it increased the number of surviving neonatal neurons.

Only two studies so far have demonstrated the potential anti-inflammatory role of sildenafil in the injured neonatal brain. Sildenafil (10 mg/kg) administered intraperitoneally has been proved to reduce reactive astrogliosis and macrophage/microglial activation at 72 h and 7 days after hypoxia-ischemia at P7 in Sprague-Dawley rats (Charriaut-Marlangue et al., 2014). Also, Moretti et al. (2016) demonstrated that a single dose of 10 mg/kg of sildenafil (10 mg/kg) administered intraperitoneally 5 min after hypoxia-ischemia at P9 reduced the recruitment and the activation of microglia in the penumbral tissue at 72 h and 8 days after injury. Interestingly, sildenafil appeared to have a distinct effect on the polarization of microglia—depending on the timing of administration compared to the timing of disease—since it functioned as an anti-inflammatory at 72 h post-HI by increasing M2-like genes expression and decreasing M1-like genes expression; and then functioned as a pro-inflammatory at 8 days post-HI by increasing M1-like genes expression and decreasing M2-like genes expression (Moretti et al., 2016).

## Effects of Sildenafil on Myelination

### Evidence in Adults

Myelin, the multilaminar sheath around axons produced in the central nervous system by oligodendrocytes (OLs), is important not only for the rapid conduction of action potential, but also for providing trophic support for axons (Nave, 2010a,b). White matter injuries (WMI) are characterized by the loss of mature OLs, the maturation arrest of pre-OLs, the degeneration of myelin, the failure of OLs migration, and the failure of myelination regeneration (Segovia et al., 2008; Huang Z. et al., 2009). The reaction of OLs varies according to the severity of the injury and its nature (acute vs. acute), and the maturity of brain development at time of injury (Emery, 2010).

It has been reported that neural stem cells (NSC) generation in rodents declines with aging due to the decrease in the basal levels of cGMP and the increase in PDE5 activity over time (Ahlenius et al., 2009). Also, in oligodendroglial precursor cells, sildenafil appeared to inhibit myelination *via* exerting a negative impact on the intrinsic oligodendroglial differentiation processes (Muñoz-Esquivel et al., 2019). However, based on other studies, sildenafil appears to be mostly protective for white matter injury. Benjamins and Nedelkoska (2007) and Yoshioka et al. (2000) found that sildenafil treatment of OLs culture promoted the survival of differentiated OLs. In a mouse model of multiple sclerosis, Pifarre et al. (2011) reported that sildenafil administered orally at peak disease could prevent axonal loss and promote remyelination with an increased number of axons with a remyelinating appearance. The administration of sildenafil from disease onset prevented OLs death at different stages of differentiation (including immature and mature myelinating OLs), preserved axons and myelin, and thus prevented disease progression (Pifarré et al., 2014). Sildenafil not only directly promoted pre-OLs maturation, but also indirectly cleared demyelinated myelin debris *via* the regulation of the microglia/macrophage inflammatory phenotype (Díaz-Lucena et al., 2018). Nunes et al. (2012) also found that sildenafil inhibited the apoptosis of OLs and maintained the normal structure of myelin and axons, which is characterized as the typical myelin lamellar arrangement with only rare fibers with reduced myelin thickness and an absence of collapsed myelin. This effect was more prominent in the group of mice receiving sildenafil from day 0–30 after injury compared to the group receiving it from day 15 to 30 (Nunes et al., 2016a), which suggests that an earlier administration after initial injury was more efficient. Sildenafil also has been proved to alleviate WMI induced by ischemia. In a mouse model of adult stroke, sildenafil promoted oligodendrogenesis by amplifying nestin-expressing NSC and their differentiation into neuronal and OLs progenitor cells that could migrate to the ischemic boundary and become mature oligodendrocytes (Zhang et al., 2012). After treatment with sildenafil, the myelin damage in iNOS-/- mice was reversed, the number of oligodendrocytes’ increased and myelin integrity and ultrastructure improved (Rapôso et al., 2014). The role of sildenafil on oligodendrocytes by balancing demyelination and remyelination appears to be independent of its anti-inflammatory effects. Any similar effects of sildenafil treatment in human adults with demyelinating diseases remains to be explored.

### Evidence in Neonates

The neonatal immature brain differs from the adult mature brain due to the rapid differentiation of immature oligodendrocytes into mature oligodendrocytes and the ongoing active myelination during the first postnatal weeks (Andersen, 2003). Shrivastava et al. (2012) have reported subcortical white matter damage and long-term atrophy related to the loss of the immature oligodendrocytes in the tracts and the loss of the subventricular zone (SVZ) OLs progenitors persisting from 3 h to 100 days after hypoxia-ischemia at P7. The vulnerability of oligodendrocytes to neonatal hypoxia-ischemia appears to be dependent on the timing of the injury related to the stage of differentiation of the oligodendrocytes (Alix et al., 2012), with the pre-OLs being markedly more susceptible to hypoxia-ischemia than the mature OLs (Back and Rosenberg, 2014).

So far, published research is lacking on the effects of sildenafil on myelination in neonates. However, the upregulation of cGMP has been shown to increase oligodendrogenesis in the developing white matter and cortex of uninjured P7 rats (Duy et al., 2015). Thus, the potential oligodendrogenesis effects of sildenafil should be further investigated in neonates.

## Effects of Sildenafil on the Vascular System

### Evidence in Adults

PDE5—the target of sildenafil—is widely distributed in cerebral vasculature, including in the smooth muscle and endothelium of the major cerebral arteries, in the brain capillary endothelial cells, and in the cerebral microvessel pericytes, a key component in the control of microcirculation and the blood-brain barrier (Kruuse et al., 2005), which explains the multiple described effects of sildenafil on cerebral vasculature, including increasing cerebral blood flow (Zhang et al., 2006; Kruuse et al., 2012), promoting microvascular reactivity (Han et al., 2012), promoting angiogenesis, etc. Increasing cerebral blood flow, resulting from either a recanalization of occluded arteries or angiogenesis, could improve the regional cerebral tissue microenvironment, which may lead to improving neurologic function recovery (Slevin et al., 2000). In a rat model of embolic stroke, a 7-day administration of sildenafil enhanced angiogenesis and increased local cerebral blood flow at 4–5 weeks after hypoxia-ischemia (Ding et al., 2008, 2011). In a rat model of epilepsy, sildenafil enhanced expression of the endothelial cell marker CD34 and the vascular endothelial growth factor (VEGF) 21 days after injury (Tawfik et al., 2018). In a rat model of Alzheimer disease, sildenafil decreased histopathological changes by modulating the vascular endothelial growth factor and the vascular cell adhesion molecule-1 (Ibrahim et al., 2021).

This vascular effect of sildenafil also has been well reported in human patients. In patients with pulmonary hypertension, sildenafil has reduced pulmonary arterial pressure and vascular resistance (Rosengarten et al., 2006). In neurologically healthy patients, sildenafil improved cerebral microvascular reactivity (Diomedi et al., 2005). In addition, a single dose of sildenafil improved the cerebral hemodynamic and increased the cerebral oxygen metabolism of patients with Alzheimer’s disease (Sheng et al., 2017). Sildenafil also attenuated injury by improving the cerebrovascular reactivity of patients with Becker muscular dystrophy (Lindberg et al., 2017). In patients with subarachnoid hemorrhage, sildenafil promoted neurofunction recovery by reducing cerebral vasospasm (Washington et al., 2016). In a randomized, double-blind, crossover, placebo-controlled trial with patients with small vessel disease (SVD), sildenafil led to a reduction in cerebral pulsatility and increased cerebrovascular reactivity, which suggests the potential benefit of sildenafil to prevent the progression of SVD (Webb et al., 2021).

### Evidence in Neonates

Again, current research on effects of sildenafil on the vessels of the neonatal brain is very limited. Notwithstanding, Charriaut-Marlangue et al. (2014) have demonstrated that sildenafil treatment can significantly increase cerebral blood flow after hypoxia-ischemia at P7. Moretti et al. (2016) have found that the NO/cGMP pathway plays a critical role in neovascularization. In addition, in growth-restricted fetal sheep, maternal treatment with sildenafil improved vascular function (Inocencio et al., 2019). The only cerebral example related to human neonates was a study that showed that sildenafil promoted cerebral oxygenation in infants after they had cardiac surgery by increasing cerebral blood flow (Nagdyman et al., 2006).

## The Influence of Sex on the Brain Effects of Sildenafil

Several studies have suggested that sex hormones may influence the effects of sildenafil on the brain. Female mice performed significantly better than males after sildenafil treatment following HI insult, which suggests a sex-dependent effect of sildenafil (Engels et al., 2017). The underlying mechanism might be related to the relationship between sex hormones and the NO/cGMP pathway. Generally, researchers have assumed that androgens positively regulate NO synthesis, and therefore, cGMP formation (Baba et al., 2000), but other research has demonstrated that androgens also upregulate PDE5, which is responsible for cGMP degradation (Morelli et al., 2004), suggesting that androgens could upregulate both cGMP formation (effect on NO) and degradation (effect on PDE5). In castrated rats, testosterone has been found to positively regulate PDE5 expression and the responsiveness to the PDE5 inhibitor (Zhang X. et al., 2005). Spitzer et al. (2013) have reported that sildenafil could increase serum testosterone in human patients by direct action on the testes. Also, Aversa et al. (2008) found that hypogonadal patients were initially resistant to sildenafil therapy and became sensitive after appropriate testosterone supplementation. Estrogen also has been demonstrated to stimulate cGMP synthesis, and the vascular protective effects of estrogen were found to be related to its influence on the NO/cGMP synthetic pathway (Sasaki et al., 2014). In a rat model of heart disease, ovary removal induced a failure of anti-remodeling properties that could be restored with estrogen replacement therapy (Sasaki et al., 2014). In ovariectomized mice, estradiol exerted an anti-depressant effect (Heydarpour et al., 2013; Saravi Seyed et al., 2017). However, the gender differences related to the neuroprotective and neurorestorative effects of sildenafil remain to be evaluated.

## Pharmacokinetics

### General Data

Sildenafil is metabolized mostly through hepatic metabolism by the CYP3A4 enzymes, and to a lesser extent by the CYP2C enzyme (Jackson et al., 1999). Sildenafil has a half-life of 0.4 h in adult rodents and approximately 4 h in human adults (Daugan et al., 2003). One mg/kg orally leads to a C_*max*_ (after 1.5 h) of 516 nM in human plasma.^1^ Sildenafil crosses the blood brain barrier (BBB) (Gómez-Vallejo et al., 2016) to reach the central nervous system disease. Gómez-Vallejo et al. (2016) have shown sildenafil to reach a concentration in cerebrospinal fluid (CSF) high enough (6–8 nM) to achieve PDE5 inhibition (the concentration required to inhibit 50% of PDE5 activity is 3.5 nM).

### Specificities of Neonates

Similar to adults, the metabolic clearance of sildenafil in neonates is catalyzed predominantly by two cytochrome P450 (CYP) isozymes: CYP3A4 (major route) and CYP2C9 (minor route) (Hyland et al., 2001); in neonates, CYP3A7 also can contribute to N-demethylation (Takahiro et al., 2015). *In vitro* studies have demonstrated a rapid maturation and increase in the expression of these enzymes immediately after birth from very low levels in the fetal liver to values similar to those in adults by the end of the first week of life, which explains why the clearance of sildenafil increased threefold from the first day after birth to the end of the first week of life (Mukherjee et al., 2009; Steinhorn et al., 2009). This suggests that the sildenafil pharmacokinetic will vary during the first week of life (Koukouritaki et al., 2004). In addition, the volume distribution of sildenafil was fourfold higher in neonates compared to adults, which resulted in a longer terminal half-life in neonates (48–56 h) compared to adults (Mukherjee et al., 2009). Recently, a population pharmacokinetics of sildenafil in extremely premature infants (≤28 weeks gestation and <32 weeks of gestation) demonstrated that oral bioavailability following enteral administration was lower in premature infants (29%) than in adults (41%) (Nichols et al., 2002). Thus, additional pharmacokinetic studies would be needed in human neonates with neonatal encephalopathy to understand optimal treatment doses, especially considering that these neonates may have liver failure and multiorgan dysfunction in addition to their neonatal encephalopathy as a result of their birth asphyxia.

## Conclusion

The current literature strongly supports the potential efficacy of sildenafil for the treatment of brain injury in adults and neonates, and also suggests that sildenafil could be both neuroprotective and neurorestorative by improving function, reducing infarct size, protecting neurons, regulating neuroinflammation, promoting myelination, and balancing vascular function. Sildenafil already is used to safely treat neonates with persistent pulmonary hypertension, and thus, it may be an ideal candidate to test with human neonates at risk of hypoxic-ischemic brain injury. However, before it can be used widely with neonates with neonatal encephalopathy, phase Ib studies are needed to prove feasibility, assess safety, and determine optimal doses and timing before assessing potential efficacy in phase II and III trials.

## Author Contributions

YX and PW contributed to conception and design of the study. YX reviewed the literature and wrote the first draft of the manuscript. PW supervised YX and revised the manuscript. Both authors contributed to manuscript revision, read, and approved the submitted version.

## Conflict of Interest

The authors declare that the research was conducted in the absence of any commercial or financial relationships that could be construed as a potential conflict of interest.

## Publisher’s Note

All claims expressed in this article are solely those of the authors and do not necessarily represent those of their affiliated organizations, or those of the publisher, the editors and the reviewers. Any product that may be evaluated in this article, or claim that may be made by its manufacturer, is not guaranteed or endorsed by the publisher.

## Footnotes

1
http://www.accessdata.fda.gov/drugsatfda_docs/NDA/98/viagra/pharm_tox_pp_117_114.pdf
